# Imaging the Acceptor Wave Function Anisotropy in Silicon

**DOI:** 10.1021/acs.nanolett.5c02675

**Published:** 2025-08-21

**Authors:** Manuel Siegl, Julian Zanon, Joseph Sink, Adonai Rodrigues da Cruz, Holly Hedgeland, Neil J. Curson, Michael E. Flatté, Steven R. Schofield

**Affiliations:** † London Centre for Nanotechnology, 127386 University College London, London WC1H 0AH, U.K.; ‡ Department of Physics and Astronomy, University College London, London WC1E 6BT, U.K.; ¶ Department of Applied Physics and Science Education, Eindhoven University of Technology, Eindhoven 5612 AZ, The Netherlands; § Department of Physics and Astronomy, 4083 University of Iowa, Iowa City, Iowa 52242, United States; ∥ Department of Electronic and Electrical Engineering, 4919 University College London, London WC1E 6BT, U.K.

**Keywords:** scanning tunneling microscopy, acceptor states, silicon, effective-mass theory, tight binding Green’s functions, quantum device engineering

## Abstract

We present the first scanning tunneling microscopy (STM) image of hydrogenic acceptor wave functions in silicon. These acceptor states appear as square-ring-like features in STM images and originate from near-surface defects introduced by high-energy bismuth implantation into a silicon (001) wafer. Scanning tunneling spectroscopy confirms the formation of a p-type surface. Effective-mass and tight-binding calculations provide an excellent description of the observed square-ring-like features, confirming their acceptor character and attributing their symmetry to the light- and heavy-hole band degeneracy in silicon. A detailed understanding of the energetic and spatial properties of acceptor wave functions in silicon is essential for engineering large-scale acceptor-based quantum devices.

## Graphical abstract

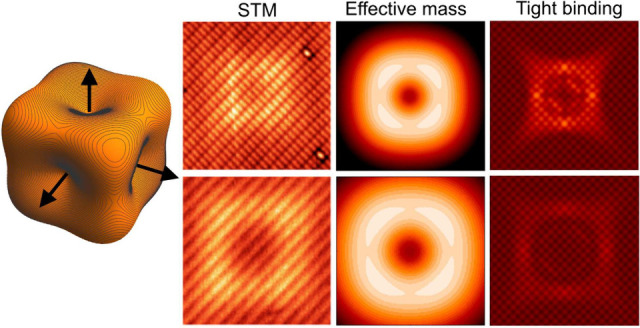

Shallow defects associated with substitutional impurities in semiconductors—known as donors when their energy levels lie near the conduction band edge or acceptors when they lie near the valence band edge—are stable and reproducible localized quantum systems. These shallow states, with binding energies of a few tens of millielectronvolts in silicon,^1^ are well described using a hydrogen-like wave function that extends over many atoms, couples to electric and magnetic fields, and provides access to both charge and spin degrees of freedom.

The spin states associated with these defects provide an exceptionally stable platform for quantum technologies, especially quantum computing. When housed in the pristine environment of isotopically purified and low defect density silicon, these spin states exhibit notably long coherence times and high resistance to noise.^2,3^ These properties, combined with the fact that each qubit is naturally identical, position dopants in semiconductors as an arguably superior choice compared to other physical qubit implementations.^4,5^

In silicon, nanometer positioning of individual phosphorus,^6^ arsenic,^7,8^ and boron^9^ donor and acceptor impurities has been achieved via scanning tunneling microscopy (STM). This has led to the fabrication of remarkable atomic-scale electronic devices, including the single-atom transistor^10^ and few-donor qubit gates.^11,12^ In order to fully utilize these devices in technologies, such as in the qubit array of an error-corrected quantum computer,^13^ the fabrication process will need to be scaled to produce millions of deterministically placed dopants with predetermined and/or gate tunable couplings. This will require not only exquisite control over the positioning of these dopants but also a detailed understanding of the spatial properties of single defects and their interactions with each other and their environment.

In compound semiconductors, like GaAs, there is a long history of dopant investigations using cross-sectional STM where the semiconductor is cleaved in situ.^14,15^ Notable successes include mapping the spatial structure of manganese^16–18^ and zinc acceptors,^19^ observing binding energy shifts as a function of the dopant depth,^20^ and manipulating the charge state of individual donors.^21^

In silicon, early measurements reported imaging the screened Coulomb potential of ionized arsenic and boron defects at room temperature using the (001) surface.^22–24^ Later, measurements of phosphorus and boron dopants at 4.2 K reported tunneling through neutral donor and acceptor states, respectively,^25,26^ and subsequent experiments observed highly anisotropic localized features attributed to the hydrogenic donor wave functions of the substitutional arsenic.^27,28^ Near-surface arsenic donors are strongly influenced by their proximity to it,^29^ but when deeper beneath the surface (∼2.5 nm), the observed features consist of a 1s-type envelope function modulated by a high-frequency Bloch component observable due to the effect of valley interference in the silicon conduction band.^30,31^

Here we present the first STM observations of hydrogenic *acceptor* state wave functions in silicon produced by high-energy bismuth implantation (see below). The observed features are large, sometimes exceeding 10 nm in lateral extent, and are highly anisotropic, exhibiting a square-shaped enhancement with edges aligned to the ⟨001⟩ directions and a central depression, giving an appearance of a “square ring” (Figure 1b,d,f). Using both multiband effective-mass approximation (EFMA) theory and atomistic tight-binding (TB) Green’s function calculations, we calculate an idealized substitutional acceptor in silicon and show that the observed anisotropy is consistent with its origin in the ground-state multiplet of a hydrogenic acceptor in silicon. Our theory takes into account both the degeneracy of the light and heavy hole bands at the Γ point as well as the local tetrahedral symmetry of the host crystal lattice.^17^ A slightly larger spatial extent of the observed features is accounted for in the EFMA theory by a small change in the effective mass of the (assumed isotropic) heavy hole band, which we attribute to strain in the near-surface region. The best fits for our EFMA and TB calculations are obtained for different acceptor depths. This discrepancy is due to the differing symmetry and accuracy of the two band structure models and impurity potential models.

**Figure 1:**
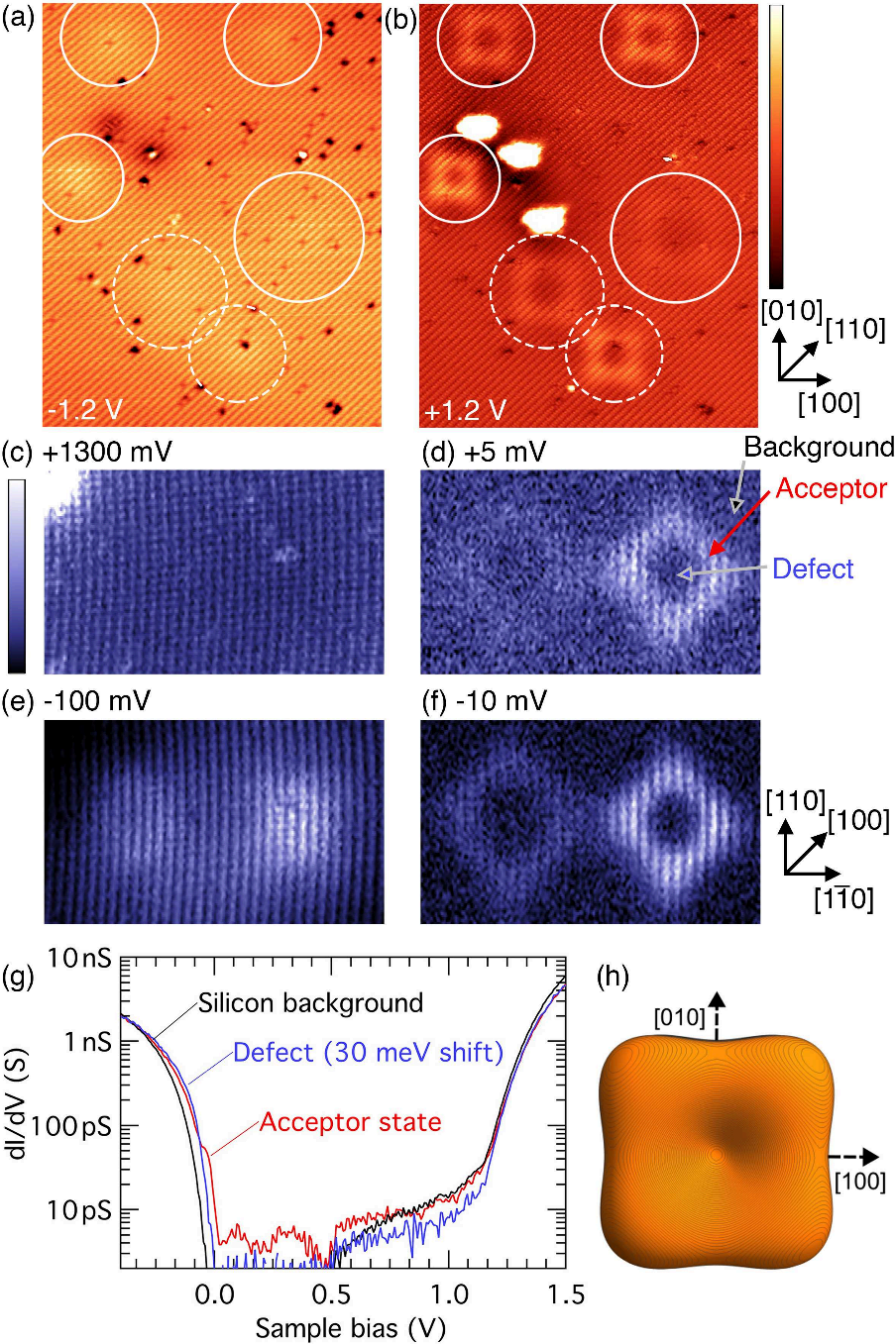
STM images of hydrogenic acceptor states beneath a Si(001) surface. (a and b) Filled- and empty-state images of the same 54 nm × 38 nm region of the surface. The locations of six acceptor states are highlighted by circles. Small black features are surface vacancy defects in the surface layer. (c–f) Differential conductance (d*I*/d*V*) maps over the two acceptors indicated by dashed circles in parts a and b at bias voltages of +1300, +5, −100, and −10 meV, respectively. (g) Spatially integrated tunneling spectroscopy showing (black) the silicon background away from the acceptor states, (red) the full region inside the square acceptor state of the rightmost acceptor, and (blue) the central depression region (only) of the rightmost acceptor. (h) Effective-mass isosurface of the probability density (|Ψ|^2^) of an ideal acceptor state in silicon, presented in more detail in Figures 2 and 3. The regulation parameters during the d*I*/d*V* measurement were −500 mV and 500 pA.

The acceptor states were created by ion-implanting a 15 Ωcm arsenic-doped Si(001) wafer to produce a bismuth density of ∼1 × 10^20^ cm^–3^ extending from 20 to 550 nm into the sample bulk (Figure S1). The sample was annealed at 500 °C overnight under ultrahigh vacuum (<2 × 10^–10^ mbar), followed by a 10 s flash anneal at 1150 °C by direct current heating, which repairs implantation damage and produces an atomically flat surface suitable for STM imaging. This bismuth implantation and annealing protocol is known to produce a high concentration of near-surface acceptor centers that persist even after thermal treatment, resulting in a p-type surface atop a deeper n-type region.^32^ While the precise microscopic structure of the observed acceptors is not known, their large spatial extent and spectral properties, described in detail below, are consistent with shallow hydrogen acceptor states.

Prior to STM imaging, the surface was passivated with hydrogen by being exposed to an atomic hydrogen beam (chamber background 5 × 10^–7^ mbar for 5 min), while the sample was maintained at 340 °C. STM images were taken at 77 K. Filled- and empty-state STM images of the same region of an implanted sample are shown in Figure 1a,b, and we have highlighted the location of six acceptor states. In the filled-state image (Figure 1a), the acceptors are barely noticeable, producing only a slight isotropic enhancement superimposed on top of the silicon dimer rows that can be seen running diagonally across the image. In contrast, the empty-state image (Figure 1b) shows very striking long-range features that are square-shaped with edges, sometimes longer than 10 nm, aligned along the ⟨100⟩ directions, and a central depression (a square ring). While each of the six acceptors has the same qualitative appearance, the width and the relative intensity of the features compared to the atomic corrugation vary from feature to feature.

Parts c–f of Figure 1 present spatially resolved conductance (d*I*/d*V*) measurements taken at the site of the two acceptors highlighted by dashed circles in Figure 1a,b (measurements at other acceptor sites exhibited similar behavior). At high positive bias (+1.3 V; Figure 1c), we resolve only the surface atomic corrugation in the d*I*/d*V* map, demonstrating that the acceptors are electrically neutral and that the conduction band states dominate the tunneling. At low positive and negative bias voltages (+5 and −10 mV; Figure 1d,f), the acceptors appear as square ring-like features. At lower negative voltage (−100 mV; Figure 1e), the acceptors appear as isotropic round elevations superimposed on the atomic lattice, indicating the band bending on the valence band states due to the negative charge of the ionized acceptors. This bias-dependent behavior, well-known from STM/STS studies of GaAs acceptors,^17^ arises from tip-induced band bending: at negative bias, the acceptor level lies below the Fermi level and donates its hole; at positive bias, it lies above and captures a hole, becoming neutral.^17,21^ The two d*I*/d*V* maps at +5 and −10 meV (Figure 1d,f) are within the substrate band gap, and therefore we do not image the silicon dimer row background surrounding the defects. Instead, we obtain a tunneling current only when directly probing the acceptor state. The acceptor state is energetically at the Fermi level, and therefore we are able to inject current into the acceptor state at low empty-state bias (Figure 1f) and extract current through the acceptor state at low filled-state bias (Figure 1d).

Figure 1g shows tunneling spectra as a function of tip bias, generated by spatially integrating the d*I*/d*V* data (i.e., the data from which the maps shown in Figure 1c–f are sampled) over different spatial regions, as indicated by arrows on Figure 1d. The black trace corresponds to the silicon background away from the acceptor states; the Fermi level lies at the valence band edge, indicating a strongly p-type surface, consistent with Hall effect measurements of samples prepared with a similar bismuth implantation procedure.^32^ Comparing this to the spectrum within the defect depression (blue trace), we observe a 30 meV upward shift of the valence band edge, confirming the defect’s negative charge state, as expected for an ionized acceptor. Extending the integration area to include the full square-shaped enhancement (red trace) reveals a pronounced shoulder near zero bias, which is the spatially resolved acceptor state seen in Figure 1d,f.

Line profiles along the [110] direction reveal the relationship between the intensity and width of the acceptor states (Figure 2a,c). Detailed analysis of 75 individual acceptors (Figure S2b,c) shows that the intensity, measured relative to the surrounding surface, decays exponentially with the feature width—consistent with probing acceptors at varying depths below the surface. We have also imaged the acceptor states as a function of bias and found that the only change is a decrease in their relative intensity compared to the background (Figure S2a). This is expected due to an increasing contribution to the tunneling current from the bulk bands. From this observation, we can rule out contributions to the appearance of the state from quasi-particle interference or Friedel oscillations.

**Figure 2:**
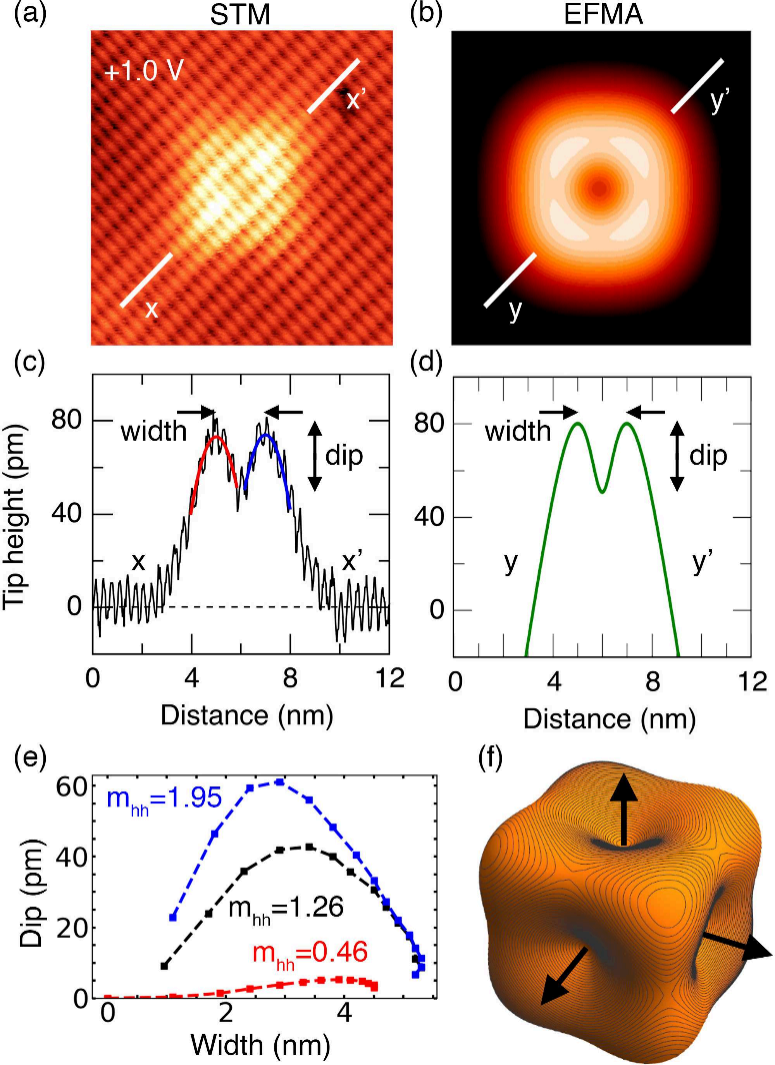
Comparison between STM measurements and EFMA theory calculations of acceptor states in silicon. (a) STM topograph acquired at +1.0 V and 20 pA (*z*-range 160 pm) showing a square ring-like feature. The white line indicates the line profile shown in part c. (b) EFMA density plot of the defect wave function, calculated for an acceptor 2.9 nm below the image plane along the [001] direction. The color range corresponds to 80 pm. (d) Line profile extracted from the effective-mass model in part b for direct comparison to the experimental data in part c. For the purpose of quantifying the features, we define the parameters “width” and “dip”, as shown in parts c and d. Both experiment and theory exhibit a dip of approximately 30 pm and a width of 2.0 nm. (e) Simulated dip versus width curves for different heavy-hole effective masses (*m*_*hh*_), with each point corresponding to a (001) plane spaced by 1 nm between 1.5 and 15.5 nm depth. (f) Isosurface of the probability density (|Ψ|^2^) at 10^–3^ nm^–3^, generated by scaling the probability density by *e* ^*qr*^, where *q* is analogous to an inverse Bohr radius (see the Supporting Information). The results in parts b and f were obtained using *m*_*lh*_ = 0.16, *m*_*hh*_ = 1.261 (β = 0.127), and κ = 1.15 Å^–1^.

The square-ring wave function described above is indicative of multiple Bloch states being involved in the description of the defect state, necessitating the use of multiband theories such as EFMA and TB Green’s functions.^17,33^ The effective-mass modeling presented here used the Luttinger Hamiltonian within the spherical approximation, with the impurity described by a zero-range potential.^34,34^ As explained in the Supporting Information (Section 2.1), our calculations obtain the cubic-symmetric solutions for the 4-fold symmetric ground state (*J* = ^3^/_2_) multiplet of the acceptor. These four solutions are described by the (degenerate) binding energy *E*_0_ and four wave functions $\Psi_{M}^{3/2}=\Psi_{M}^{3/2}\left(r,\theta ,\varphi \right)$ , where *M* labels are ±^3^/_2_ and ±^1^/_2_. The 4-fold degeneracy of the multiplet will produce a measurement of the wave function that is a temporal average of the four *M* states. Because time-reversal symmetry requires the two light-hole states (±^1^/_2_) to have identical local densities of states and likewise two heavy-hole states (±^3^/_2_), we expect

1 $${\left|\Psi_{avg}\right|}^{2}=\frac{1}{2}(|\Psi_{1/2}^{3/2}{|}^{2}+|\Psi_{3/2}^{3/2}{|}^{2})$$

Assuming a spherically symmetric delta-function-like potential, centered at *r* = 0, and the averaging described in eq 1, the analytic solution for the defect wave function is

2 $${\left|\Psi_{\mathrm{avg}}\left(r,\theta ,\varphi \right)\right|}^{2}=\frac{{\left|R_{0}\left(r\right)\right|}^{2}}{16\pi }+5f\left(\theta ,\varphi \right)\frac{{\left|R_{2}\left(r\right)\right|}^{2}}{16\pi }$$

where *R*_0_(*r*) and *R*_2_(*r*) are radial functions that depend on the binding energy *E*_0_ and the mass ratio β = *m*_*lh*_/*m*_*hh*_, where *m*_*lh*_ and *m*_*hh*_ are the light- and heavy-hole effective masses at the Γ point. The presence of the angular function *f*(θ,φ) in the second term of eq 2 causes |Ψ_avg_(*r*,θ,φ)|^2^ to exhibit a d-like orbital character.

To make comparisons with the STM measurements, it is useful to consider the relationship among the tunneling current, the local density of states, and the defect wave function. The tunneling current reflects the probability of an electron transferring between the STM tip and the sample through vacuum and depends sensitively on both the local density of states and the tip-sample separation. In the simplest case, assuming a delta-function tip with a constant density of states, the tunneling current is given by

3 $$I\left(\omega ,r,\theta ,z\right)∼\eta \left(\omega ,r,\theta ,0\right)e^{-2\kappa z}$$

where *z* is the tip-sample separation, ω is the energy corresponding to the applied bias, and κ is the decay constant of the tunneling current. When on resonance with the defect, the sample local density of states η is dominated by the defect and is well approximated by η = |ψ|^2^. Solving for the STM height expressed as a function of the defect probability density yields

4 $$\mathrm{Height}\left(r,\theta ,\varphi \right)=\frac{1}{2\kappa }\;\ln\left(\frac{{\left|\Psi_{\mathrm{avg}}\left(r,\theta ,\varphi \right)\right|}^{2}}{{\left|\Psi_{\mathrm{avg}}\left(r_{0},\theta_{0},\varphi_{0}\right)\right|}^{2}}\right)$$

where we have included an offset to set the wave function located at *r*_0_ to correspond to Height = 0.

In Figure 2b,d–f, we show an EFMA wave function calculated considering the impurity site 2.9 nm below the (001) cleaved plane, using *E*_0_ = 30 meV, *m*_*lh*_ = 0.16, and *m*_*hh*_ = 1.261 (β = 0.127). This produces excellent agreement between our theoretical results and the experimentally observed feature shown in Figure 2c,d, having a peak-to-peak width of 2 nm and a dip of 30 pm. Our effective-mass model in Figure 2f presents clearly a symmetry similar to that reported in ref 35 for an excited state of an acceptor in Si; here, however, we show that this structure emerges for the ground state.

The optimal values for β and the [001] plane depth were obtained by using Figure 2e. For κ = 1.15 Å^–1^ and the light-hole mass fixed at its bulk value (*m*_*lh*_ = 0.16), Figure 2e shows that increasing the heavy-hole mass *m*_*hh*_ enhances both the feature dip and lateral width of the simulated line profile. The deviation of *m*_*hh*_ from its bulk value likely reflects the influence of the surface strain field on the hole dispersion; the strain-induced splitting of bulk valence bands has a well-understood effect on the valence band dispersion and anisotropy.^1^ In addition to this, it is well-known that, among tetrahedral semiconductors, Si has a substantial anisotropy for the hole masses (larger, for example, than gallium arsenide);^36^ therefore, the optimal β = 0.127 value is an isotropic value that approximates this anisotropy. A more detailed discussion of the parameter choices, including the hole masses and tunneling decay constant κ, is provided in the Supporting Information (Section 2.1).

As the number of atomic planes from the impurity increases, corresponding in the experiment to a greater defect depth below the surface, we continue to find good qualitative agreement between the STM measurements and EFMA results. Figure 3 shows a comparison between experimental STM images (left column) and EFMA simulations (center column). The right column of Figure 3 shows corresponding atomistic TB simulations, which also exhibit excellent agreement with the STM data and are discussed further below. In the STM measurements, the top to bottom rows correspond to increasing feature width that we attribute to the defect depth beneath the surface, while in the EFMA simulations, they correspond to wave function evaluations at planes located progressively farther from the impurity site (2.9, 3.9, and 5.9 nm). The agreement between the STM and EFMA simulations is excellent and becomes increasingly better as the defect depth increases.

**Figure 3:**
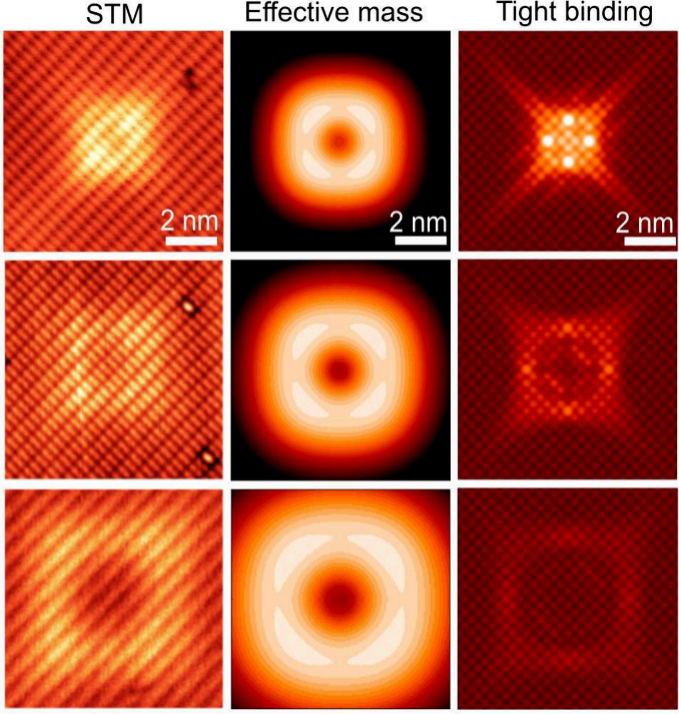
Comparison of STM measurements (left column), effective-mass modeling (center column), and TB calculations (right column) for acceptor states in silicon. STM image parameters (top to bottom): 1.1 V, 20 pA, *z*-range 160 pm; 1.6 V, 20 pA, *z*-range 30 pm; 1.0 V, 5 pA, *z*-range 100 pm. For the effective-mass results, contour plots are shown for acceptors at depths of 2.9, 3.9, and 5.9 nm below the image plane. The TB simulations show topographical scans of the acceptor local density of states at depths of 0.81, 1.62, and 2.17 nm below the surface. Both effective-mass and TB images use a color intensity scale with a range of 80 pm.

To gain an additional atomistic perspective on the electronic structure, we turn next to TB calculations. In particular, the Green’s function method used here is a many-band single-particle theory formulated on a TB basis. This method casts the defect problem in terms of bulk Bloch waves scattering off a localized defect potential. Formally, this is expressed using the Dyson equation

5 Ĝ(ω)=[1−V′^ĝ(ω)]−1ĝ(ω)

where *V*′ is the perturbative (inhomogeneous) potential associated with the defect, ĝ are bulk (unperturbed) Green’s functions, and Ĝ are the resultant inhomogeneous Green’s functions containing information about the defect resonant energy and wave function. We compute the real-space bulk Green’s functions, *g*(ω), by taking the inverse Fourier transform of the resolvent of the bulk electronic Hamiltonian

6 $$g\left(r,r^{\prime };z\right)=\int_{\mathrm{BZ}}{\left[z-Ĥ\left(k\right)\right]}^{-1}e^{i\left(r-r^{\prime }\right)\cdot k}\;dk$$

We employ an empirical spds* TB model (including s, p, d, and excited s* orbitals) for silicon that includes spin–orbit coupling.^37^ This model accurately reproduces the conduction and valence band edges and effective masses throughout the Brillouin zone.

A key detail, as it pertains to the computational cost of this method, is that if *V*′ is well described by a relatively small cluster of atoms with short-ranged interactions, then eq 5 can be efficiently and exactly solved. Expressed in block form, it can be written as

7 Ĝ=(ĜnnĜnfĜfnĜff)=(M̂nnĝnnM̂nnĝnfĝfnM̂nnĝff+ĝfnV′^nnM̂nnĝnf)

where M̂n,n=(1−ĝn,nV′^n,n)−1 . Here the subscripts “n” and “f” refer to the so-called near and far fields, as described in the Supporting Information. In eq 7, the computation of the inhomogeneous Green’s functions depends only on the local propagator (*g*_*ii*_), the propagator from the local site to the defect impurity (*g*_*i*,*n*_), and the description of the local impurity in the near-field defined by *M*_*nn*_. Because there is no coupling of terms between two differing far-field sites, the defect calculation avoids truncation effects when computing finite-size fields of view.

The local density of states, η, can be directly extracted from the inhomogeneous Green’s functions via the relationship

8 η(r;ω)=−1πIm[Tr[Ĝ(r,r;ω)]]

Topographical images for comparison with STM images are generated by computing η in conjunction with eq 4 for every atomic site in the field of view for a given plane and then convolving with a spherical Gaussian of full width at half-maximum of one-quarter the nearest-neighbor bond distance (*d*_NN_ = 2.35Å).

The right column of Figure 3 shows acceptor states generated by using this method. We found excellent agreement between our TB Green’s function calculations for a defect modeled as an ideal substitutional impurity with *T*_*d*_ site symmetry, located 30 meV above the valence band edge, and the experimentally observed acceptor state wave function. The series of images with increasing depth in Figure 3 demonstrates the evolution of the defect wave function with depth. As with the EFMA observations (center column, Figure 3), these images reproduce extremely well the features seen in the experimental data (left column, Figure 3), although we obtain slightly different predictions for the defect depth (0.81, 1.62, and 2.17 nm) compared to those in the EFMA theory.

Some features—such as the four central bright spots in the 0.81 nm depth feature (top right, Figure 3)—likely arise from neglecting near-defect relaxation of neighboring atoms. We further note that the EFMA and TB models have different strengths, which can account for the discrepancies between them. We have included a brief discussion of this in the Supporting Information and calculations of a split-vacancy configuration and why this does not match the experimentally observed features.

This work presents the first images of acceptor state wave functions in silicon. Through STM measurements, the acceptor states are identified as square ring-like features with strong electronic contrast superimposed on the atomic lattice of the Si(001) surface. Tunneling spectroscopy and spatial d*I*/d*V* conductance maps confirm their spatial structure, energetic position approximately 30 meV above the valence band edge, and role in inducing a p-type Si(001) surface. Comparison with effective-mass and atomistic TB calculations confirms that the observed features are consistent with acceptor states in silicon and provides deeper insight into their characteristics. The effective-mass model clearly reveals a d-like contribution to the square ring appearance, while the TB calculations indicate that the observed symmetry arises from a substitutional impurity with *T*_*d*_ site symmetry. The combination of a high-resolution experiment and detailed theoretical modeling demonstrated here provides the foundation for a thorough understanding of acceptor states in silicon, which is essential for future quantum technological applications based on these defects.

## Supplementary Material



## Notes

Experimental methods and results, ion implantation simulations, STM image analysis and statistics, parameter tables and figures, theoretical methods and results, effective-mass approximation, TB Green’s function, and comparison to the split-vacancy model (PDF)
