# Supplementary material for: Imaging the Acceptor Wave Function Anisotropy in Silicon
Source: Nano Lett. 2025 Aug 21;25(38):13996–4001. doi: 10.1021/acs.nanolett.5c02675 (PMC12464996; doi:10.1021/acs.nanolett.5c02675)
Supplement: Supplementary file 1 [file nl5c02675_si_001.pdf]

Supporting Information for

Imaging the acceptor wave function anisotropy in silicon

Manuel Siegl[1,2], Julian Zanon[3], Joseph Sink[4], Adonai Rodrigues da Cruz[4], Holly Hedgeland[1], Neil J. Curson[1,5], Michael Flatté[*3,4], and Steven R. Schofield[†1,2]

[1]London Centre for Nanotechnology, University College London, WC1H 0AH, London, UK
[2]Department of Physics and Astronomy, University College London, WC1E 6BT, London, UK
[3]Department of Applied Physics and Science Education, Eindhoven University of Technology, Eindhoven 5612 AZ, The Netherlands
[4]Department of Physics and Astronomy, University of Iowa, Iowa City, Iowa 52242, USA
[5]Department of Electronic and Electrical Engineering, University College London, WC1E 6BT, London, UK

June 20, 2025

This supplement material is organized as follows: Additional experimental methods and data are presented in Sec. S1., the effective mass approximation is discussed in Sec. S2.1., and the tight-binding method follows in Sec. S2.3.. The data presented in Sec. S1. is extensively discussed in Siegl's PhD thesis [5].

S1. Supplementary experimental methods and results

Ion implantation was performed at the University of Surrey using Bi^+ ions at five energies between 0.25 and 2.0 MeV, with doses ranging from 0.41 to 1.9×10^{15} cm^{-2}. The implantation parameters are listed in Table S1.

The depth profile resulting from this implantation sequence was estimated using the Stopping and Range of Ions in Matter (SRIM) simulation package [7], as shown in Supplementary Fig. S1. The simulation predicts a rapid increase in Bi concentration from the surface to approximately 1×10^{20} cm^{-3} at 20 nm depth, remaining nearly constant to a depth of 550 nm, and then decaying toward background by 700 nm.

*Email: michaelflatte@quantumsci.net
†Email: s.schofield@ucl.ac.uk

Table S1: Ion implantation parameters for bismuth in Si(001).

Energy (MeV)	Dose (cm^{-2})
2.00	1.9×10^{15}
1.35	1.2×10^{15}
0.85	0.9×10^{15}
0.50	0.64×10^{15}
0.25	0.41×10^{15}

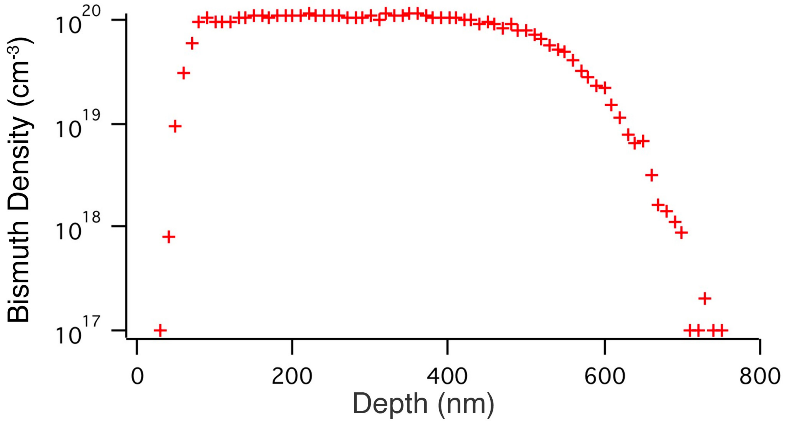

Figure S1: SRIM-estimated depth profile of Bi ion concentration for the implantation conditions listed in Table S1.

To further characterize the behavior of the acceptor states, we performed bias-dependent imaging, as shown in Supplementary Fig. S2a, which shows empty- and filled-state STM images of a single acceptor over a range of imaging biases from ±1.7 V to ±1.0 V. For each bias polarity, the overall appearance of the state remains unchanged within this bias range, with only a decrease in relative intensity compared to the background at higher biases. This behavior is consistent with an increasing contribution of bulk band states to the tunneling current and rules out interpretations based on quasiparticle interference or Friedel oscillations.

We also performed a statistical analysis of the spatial characteristics of many features. To quantify the spatial characteristics of the acceptor states, we extracted line profiles along the [110] direction, as illustrated in Supplementary Fig. S2b. The width and intensity of 75 individual acceptor states were measured and are summarized in Supplementary Fig. S2c. The data reveal an exponential decay of intensity with increasing feature width, consistent with a distribution of acceptor depths: deeper states exhibit reduced spatial overlap with the STM tip wave function and a broader lateral extent at the surface. These results support our interpretation that the variability in the observed features arises primarily from differences in the depth of the acceptor states beneath the surface.

S2. Supplementary theoretical methods and results

S2.1. Effective mass approximation (EFMA)

Considering the spherical symmetric Luttinger Hamiltonian with the impurity described by a zero-range potential, it is possible to find an analytical solution for an acceptor ground state as shown in [1]. Following [6], the spherical ground state can be used to construct the following solution

$$\Psi_{3/2}(r,\theta,\phi) = c^{\Gamma_1} R_0(r) \begin{pmatrix} Y_{0,0} \\ 0 \\ 0 \\ 0 \end{pmatrix} + \frac{c^{\Gamma_{25}}}{\sqrt{2}} R_2(r) \begin{pmatrix} 0 \\ -2Y_{2,1} \\ Y_{2,2} - Y_{2,-2} \\ 0 \end{pmatrix} + \frac{c^{\Gamma_{12}}}{\sqrt{2}} R_2(r) \begin{pmatrix} \sqrt{2}Y_{2,0} \\ 0 \\ Y_{2,2} + Y_{2,-2} \\ 0 \end{pmatrix}, \tag{1}$$

$$\Psi_{1/2}(r,\theta,\phi) = c^{\Gamma_1} R_0(r) \begin{pmatrix} 0 \\ Y_{0,0} \\ 0 \\ 0 \end{pmatrix} + \frac{c^{\Gamma_{25}}}{\sqrt{2}} R_2(r) \begin{pmatrix} 2Y_{2,1} \\ 0 \\ 0 \\ Y_{2,2} - Y_{2,-2} \end{pmatrix} + \frac{c^{\Gamma_{12}}}{\sqrt{2}} R_2(r) \begin{pmatrix} 0 \\ -\sqrt{2}Y_{2,0} \\ 0 \\ Y_{2,2} + Y_{2,-2} \end{pmatrix}, \tag{2}$$

which now has a cubic symmetry such as the top of the valence bands in a zincblende crystal. Note that, because of time-reversal symmetry, the states $\Psi_{-3/2}$ and $\Psi_{-1/2}$ are Kramers conjugates of $\Psi_{3/2}$ and $\Psi_{1/2}$, therefore they are not shown here explicitly. The spherical harmonics terms

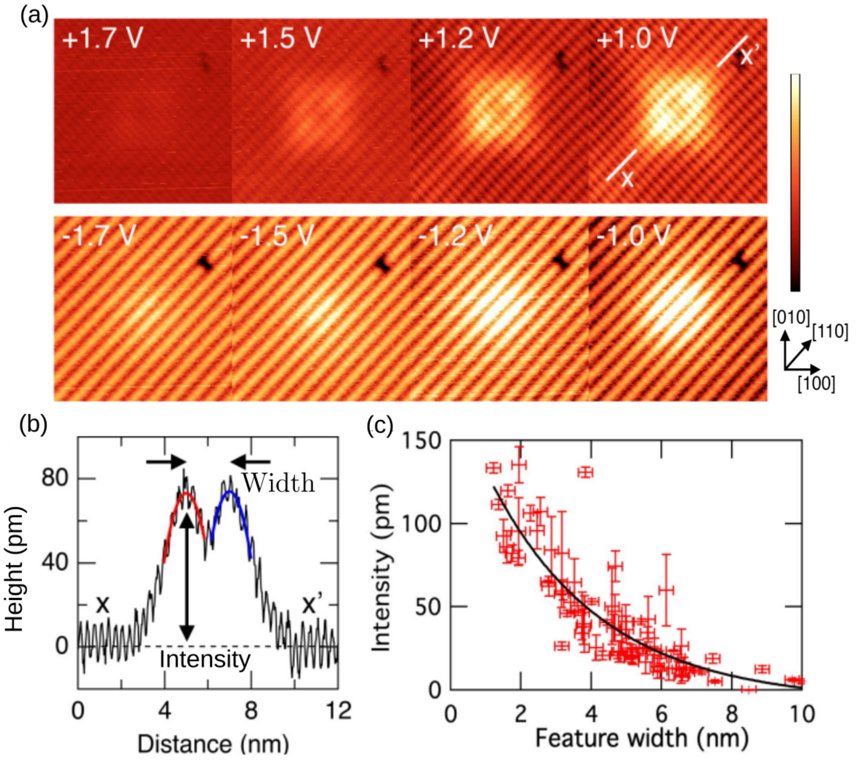

Figure S2: (a) Empty and filled-state images ($10{\times}10$ nm^2) of a single acceptor over a range of imaging bias from ±1.7 to ±1.0 V. Image set point, 20 pA. The imaging set point was 20 pA. The z-range is 150 pm for the empty-state images and 80 pm for the filled-state images. (b) Example line profile over a single feature illustrating how the feature intensity and width are extracted. (c) Plot of relative intensity versus width for 75 acceptor states. The images were recorded with tunneling bias in the range $+0.9$ to $+1.2$ V, and with different tips, resulting in a ±10 pm contribution to the uncertainty of the intensities.

$Y_{l,m} \equiv Y_{l,m}(r,\theta,\phi)$ that go along with the coefficients c^{Γ_1}, $c^{\Gamma_{25}}$ and $c^{\Gamma_{12}}$ transform like Γ_1(s-like), Γ_{12} ($d_{x^2-y^2}$ - like) and Γ_{25} (d_{xy} - like), respectively. In analogy what is done in [6], we consider $c^{\Gamma_1} = 1$ and the coefficients $c^{\Gamma_{25}}$ and $c^{\Gamma_{12}}$ are parameterized by η (with $0 \leq \eta \leq 1$) as

$$c^{\Gamma_{25}} = \frac{1}{\sqrt{3+2\eta^2}}, \; c^{\Gamma_{12}} = \frac{\eta}{\sqrt{3+2\eta^2}} \; . \tag{3}$$

For $\eta = 0$ ($\eta = 1$) the solution above has a cubic (spherical) symmetry. The radial functions in Eqs.(1) and (2) have the following form

$$R_0(r) = \frac{C_0}{r}\left[\beta\, e^{-qr\sqrt{\beta}} + e^{-qr}\right], \tag{4}$$

$$R_2(r) = \frac{C_0}{r}\Big[-\beta\, e^{-qr\sqrt{\beta}}\left(1 + \frac{3}{qr\sqrt{\beta}} + \frac{3}{q^2r^2\beta}\right) + e^{-qr}\left(1 + \frac{3}{qr} + \frac{3}{q^2r^2}\right)\Big] . \tag{5}$$

with the constants C_0, β and q determined by

$$C_0 = \sqrt{\frac{q}{\beta\sqrt{\beta}+1}}, \; \beta = \frac{m_{lh}}{m_{hh}}, \; q = \sqrt{\frac{2\, m_{hh} E_0}{\hbar^2}}, \tag{6}$$

where m_{lh} and m_{hh} are the light and heavy hole masses determined within the spherical approximation for the Luttinger parameters, m_0 is the electron mass and E_0 is the binding energy.

The average solution that represents the acceptor states measured with STM is

$$\left|\Psi_{\text{avg}}\right|^2 = \frac{1}{2}\left(\left|\Psi_{1/2}\right|^2 + \left|\Psi_{3/2}\right|^2\right), \tag{7}$$

and using Eqs.(1) and (2), we find

$$\begin{aligned} \left|\Psi_{\text{avg}}(r,\theta,\phi)\right|^2 = {} & \left|R_0(r)\right|^2 \left|Y_{0,0}(\theta,\phi)\right|^2 + \left|R_2(r)\right|^2 \Big[\frac{\eta^2}{(3+2\eta^2)}\left|Y_{2,0}(\theta,\phi)\right|^2 + \frac{2}{(3+2\eta^2)}\left|Y_{2,1}(\theta,\phi)\right|^2 \\ & + \left(\frac{(\eta^2+1) + (\eta^2-1)\cos(4\phi)}{3+2\eta^2}\right)\left|Y_{2,2}(\theta,\phi)\right|^2\Big] \; . \end{aligned} \tag{8}$$

Observe that, for $\eta = 1$ the Eq.(8) reduces to spherical solution given by

$$\left|\Psi_{\text{avg}}(r)\right|^2 = \frac{1}{4\pi}\left(\left|R_0(r)\right|^2 + \left|R_2(r)\right|^2\right) \tag{9}$$

In contrast, for $\eta = 0$, Eq.(8) has an angular dependency that multiplies the $R_2(r)$ term

$$\left|\Psi_{\text{avg}}(r,\theta,\phi)\right|^2 = \frac{1}{4\pi}\left|R_0(r)\right|^2 + \frac{5}{4\pi}\left|R_2(r)\right|^2 \left[\cos^2(\theta)\sin^2(\theta) + \sin^2(\phi)\cos^2(\phi)\sin^4(\theta)\right] \; . \tag{10}$$

Graphically, the solutions for the spherical $\eta = 1$ and cubic $\eta = 0$ given by Eqs.(9) and (10), respectively, are shown in Supplementary Fig.S3.

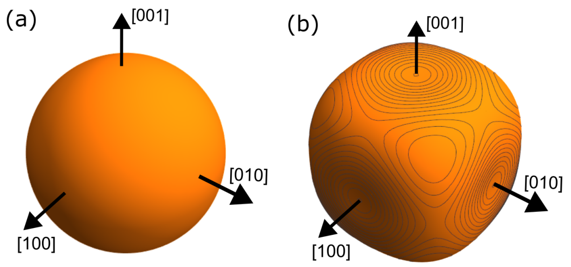

Figure S3: a) and b) show $|\Psi_{\rm avg}|^2\, e^{qr} = 5\times10^{-4}\,\rm cm^{-1}$ for $\eta = 1$ and $\eta = 0$, respectively. Note that when $\eta \to 0$ the spherical symmetry is no longer present.

Table S2: Parameters used for plots in Supplementary Figs. S3 and S4. m_{lh} and m_{hh} are calculated considering the spherical approximation for the Luttinger parameters, and E_0 is the expected binding energy for the ground-state acceptor in silicon (Si).

Material	γ_1	γ_2	γ_3	m_{lh}/m_0	m_{hh}/m_0	β	E_0 (meV)
Si	4.22	0.39	1.44	0.159	0.458	0.348	30

The experimental images show strong electronic contrasts with a square ring-like feature at planes along the [001] direction, through the height from the tip to the surface. In analogy to this, we use the following relation

$$\text{Height}(r,\theta,\varphi) = \frac{1}{2\kappa}\ln\left(\frac{\left|\Psi_{\rm avg}(\mathrm{r},\theta,\varphi)\right|^2}{\left|\Psi_{\rm avg}(\mathrm{r}_0,\theta_0,\varphi_0)\right|^2}\right) . \tag{11}$$

Here $(r_0, \theta_0, \varphi_0)$ denotes the point at which Height $= 0$ and κ is the tunnel decay length constant, which usually in STM measurements is ~ 1 Å^{-1}.

S2.2. Supplementary EFMA results

Supplementary Fig.S4 presents the Height(r, θ, φ), Eq.(11), using parameters from Tab.S2. The effective mass result in Fig. S4a shows qualitatively a 'square ring' shape, similar to the STM image in Fig.2 present in the paper. However, comparing the Height profile in Fig. S4b with the profile obtained from STM (Fig.2 from the main text), the width and dip from the calculations are off from the experimental results.

To obtain the results in Fig.S4, the binding energy used was $E_0 = 30$ meV based on the STM results. A similar value is found from the shallow-state solution of the Luttinger-Kohn Hamiltonian for a Coulomb potential [2]. The value for β assumes the spherical approximation for the Luttinger parameters, see Tab.S2. However, the actual Si samples have a terminating (001) surface at which the STM measurements are performed; surfaces can cause the appearance of strain [4] which may change the light- and heavy-hole masses m_{lh} and m_{hh}, respectively.

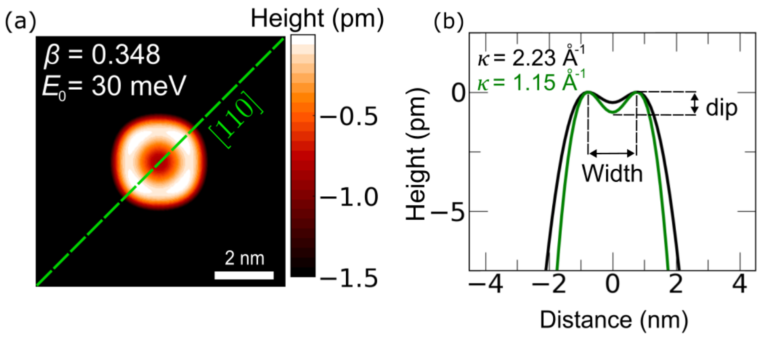

Figure S4: a) shows the height at a (001) plane 5 nm distant from the impurity site, the green line indicates the [110] direction of the profile plot in b), where width = 1.5 nm and dip = 1.0 pm. Tab.S2 contains the parameters for these calculations. a) is qualitatively similar to the STM images, however from the profile line in (b) the 'square ring' feature does not have the same dip and width as observed from the STM profile in Fig.2 from the paper, which has dip ≈ 30 pm for a width ≈ 2 nm.

Considering the above mentioned problems, we explored our model in order to identify a possible set of optimal parameters for m_{lh} and m_{hh} that could reproduce the dip and width measured. Therefore, the Height was calculated at different planes along the [001] direction considering various values for β and κ, see Figs. S5 - S7.

Fig. S5 shows that the dip at different [001] planes acquires a peak, and its value for a given κ depends only on the ratio between light and heavy hole masses β. As β decreases from 0.348 the peak value for the dip increases. If β increases the peak value decreases, e.g., fixing $m_{hh} = 0.688$ and increasing m_{lh} from 0.08 in Fig.S6a to 0.319 in Fig.S6d we clearly see the peak value decreasing. Something similar happens if m_{lh} is fixed (e.g., $m_{lh} = 0.16$) and m_{hh} decreases, check Fig.S6b.

Fig. S6 presents a similar behavior for the width. For a given κ, as β decreases from 0.348, the peak value for the width increases. Furthermore, as β decreases, the peak value moves closer to the impurity site.

To find an optimal value for m_{lh}, m_{hh} and κ, we need to combine the results from Figs. S6 and Fig. S6, such that for a given width we have its respective dip. Therefore Fig.S7 summarizes our analyses, with dip versus width at different planes. A similar image is shown Fig.2 in the paper, where $m_{lh} = 0.16$ (i.e., its bulk value) and after varying m_{hh} we find that $m_{hh} = 1.261$ for a plane 2.9 nm deeper from the impurity site, produce a square-ring feature with dip= 30 pm and width= 2.0 nm, similar to the experimental values. m_{lh} was fixed based on the idea that the light-hole mass is likely less affected by strain than the heavy-hole mass. For example, in Ref. [3] the Luttinger-Kohn Hamiltonian was solved for heterostructures under strain, and m_{lh} was found to be less susceptible to strain than m_{hh}.

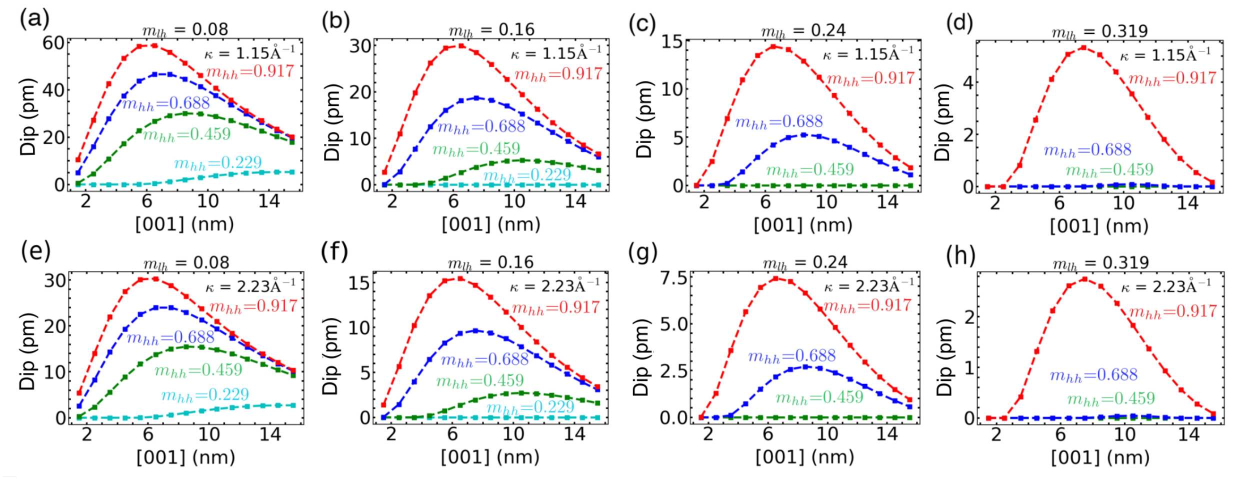

Figure S5: dip versus the plane distance [001] for different values of m_{lh} and m_{hh} (both normalized by m_0) using $\kappa = 1.15$ Å^{-1} in a), b), c) and $\kappa = 2.23$ Å^{-1} in d), e) and f).

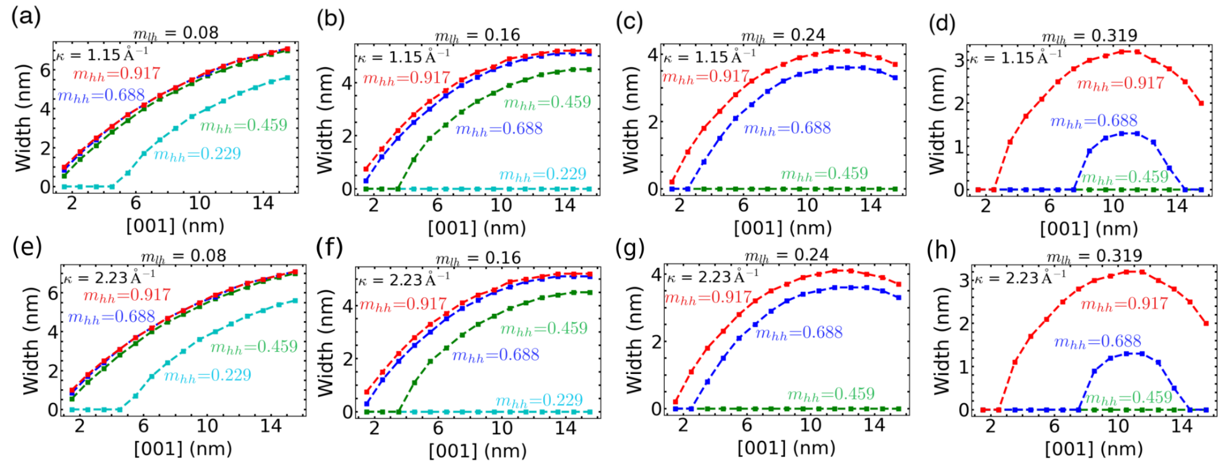

Figure S6: Width versus the plane distance [001] for different values of m_{lh} and m_{hh} (both normalized by m_0) using $\kappa = 1.15$ Å^{-1} in a), b), c) and $\kappa = 2.23$ Å^{-1} in d), e) and f).

S2.3. Tight-Binding Green's Function

For states near a band edge, a decomposition in terms of Bloch states often can be shown to have a simple dependence on a single band and a small wavenumber $\vec{k}$. As states move deeper into the band gap the range of $\vec{k}$ and the number of bands required to effectively model the state increases. In such cases, it becomes necessary to utilize multiband electronic models that are accurate over a wide range of crystal momenta, and ideally the entire Brillioun zone.

To study localized point defects, we solve the Dyson equation,

$$\hat{G}(\omega) = (1 - \hat{V}'\hat{g}(\omega))^{-1}\hat{g}(\omega) \tag{12}$$

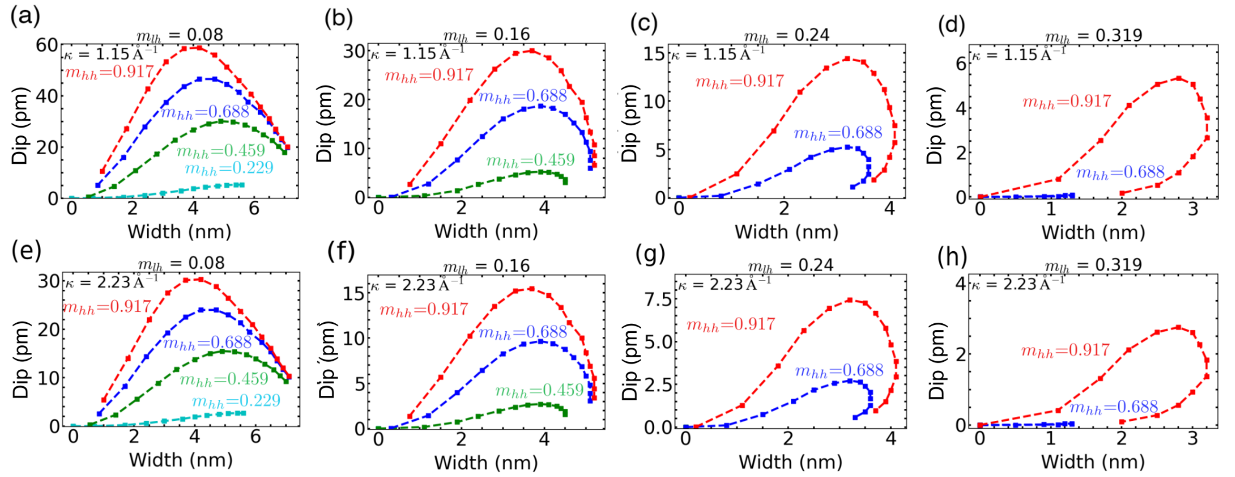

Figure S7: dip versus width for different values of m_{lh} and m_{hh} (both normalized by m_0) using $\kappa = 1.15$ Å^{-1} in a), b), c) and $\kappa = 2.23$ Å^{-1} in d), e) and f)..

for the inhomogeneous Green's functions, which correspond to the amplitudes with which bulk Bloch functions scatter off of the localized defect potential, V'.

The real-space bulk Green's functions, $g(\omega)$, are computed by taking the numerical inverse Fourier transform of the resolvent of the reciprocal-space Hamiltonian,

$$g(\mathbf{r}, \mathbf{r}'; z) = \int_{BZ} [z - \hat{H}(\mathbf{k})]^{-1} e^{i(\mathbf{r}-\mathbf{r}')\cdot\mathbf{k}} d\mathbf{k} \tag{13}$$

where $z = \omega + i\delta$. The small imaginary contribution from δ is necessary to avoid the singularity that arises when $\omega = E(\mathbf{k})$, where $E(\mathbf{k})$ is the energy dispersion found by diagonalizing $H(\mathbf{k})$. The δ value corresponds to a minimum line-width that limits the maximum resolution obtained for spectral features in the calculation.

For a defect potential that extends over a finite range of atoms in the crystal, the Dyson equation can be solved exactly by separating the problem into two parts, namely, the so-called "near field" and "far field". The near field is defined to contain all atoms where the defect potential has a non-zero value, and the far field is defined to encompass the remainder of atoms in the calculation (where the defect potential is zero). In this basis, our operators can be expressed in block form as,

$$\hat{G} = \begin{pmatrix} \hat{G}_{nn} & \hat{G}_{nf} \\ \hat{G}_{fn} & \hat{G}_{ff} \end{pmatrix} \qquad \hat{g} = \begin{pmatrix} \hat{g}_{nn} & \hat{g}_{nf} \\ \hat{g}_{fn} & \hat{g}_{ff} \end{pmatrix} \qquad \hat{V} = \begin{pmatrix} \hat{V'}_{nn} & 0 \\ 0 & 0 \end{pmatrix} \tag{14}$$

Plugging in the block form in Equation 14 into Equation 12, we end up with an exactly solvable set

of equations for the inhomogeneous Green's functions,

$$\hat{G} = \begin{pmatrix} \hat{G}_{nn} & \hat{G}_{nf} \\ \hat{G}_{fn} & \hat{G}_{ff} \end{pmatrix} = \begin{pmatrix} \hat{M}_{nn}\hat{g}_{nn} & \hat{M}_{nn}\hat{g}_{nf} \\ \hat{g}_{fn}\hat{M}_{nn} & \hat{g}_{ff} + \hat{g}_{fn}\hat{V'}_{nn}\hat{M}_{nn}\hat{g}_{nf} \end{pmatrix} \tag{15}$$

where

$$\hat{M}_{n,n} = (1 - \hat{g}_{n,n}\hat{V'}_{n,n})^{-1} \tag{16}$$

We can see from Equation 15 that the computation of the inhomogeneous Green's functions depends only on the local propagator (g_{ii}), the propagator from the local site to the defect impurity ($g_{i,n}$), and the description of the local impurity in the near field defined by M_{nn}. As there is no coupling of terms in the far field, this method is free from artifacts introduced by methods relying on exact diagonalization, such as finite volume effects and descriptions of surface terminations.

To generate the simulated topographical scans, we first need to extract the local density of states, η, from the inhomogeneous Green's functions. η is related to the imaginary part of the Green's function via the relation

$$\eta(\mathbf{r};\omega) = \frac{-1}{\pi} Im[Tr[\hat{G}(\mathbf{r},\mathbf{r};\omega)]]. \tag{17}$$

Simulated topographical scans of defects are generated by computing η for every atomic site (i.e., $\mathbf{r}$) in the field of view at a given plane height, and then convolving with a spherical Gaussian of FWHM of one-quarter the nearest-neighbor bond distance ($d_{NN} = 2.35$Å).

S2.4. EFMA and TB comparison

We note that the EFMA and TB models have different strengths, which can account for the discrepancies between them. The simpler (isotropic bulk dispersion) effective mass model permits a straightforward modification of the valence effective masses to study the effects on the wave function shape and choose optimal parameters. The tight-binding model includes spherical anisotropy of the dispersion, however features like the effective mass depend on many parameters that also affect other properties, so adjusting one mass is very difficult. Neither model includes the distortion of the electronic structure induced by the surface, which may also have an effect on the correct masses and dispersion anisotropy.

S2.5. Supplementary tight binding Green's function results

To assess the possibility that the observed acceptor state could originate from a microscopic defect with a symmetry different from that of the silicon crystal lattice, we modeled a split-vacancy configuration characterized by S_6 site symmetry The simulated wave function for this defect exhibited strong deviations from the experimentally observed square ring-like structure. The additional calculation assumed a split-vacancy model with a bound state at 30 meV. The resulting wave function has S_6 site symmetry and can be seen in Fig. S8 to strongly deviate from the experimental square-ring spatial structure. We therefore conclude that the split-vacancy model is inconsistent with the experimental data and is an unlikely candidate for the observed defect.

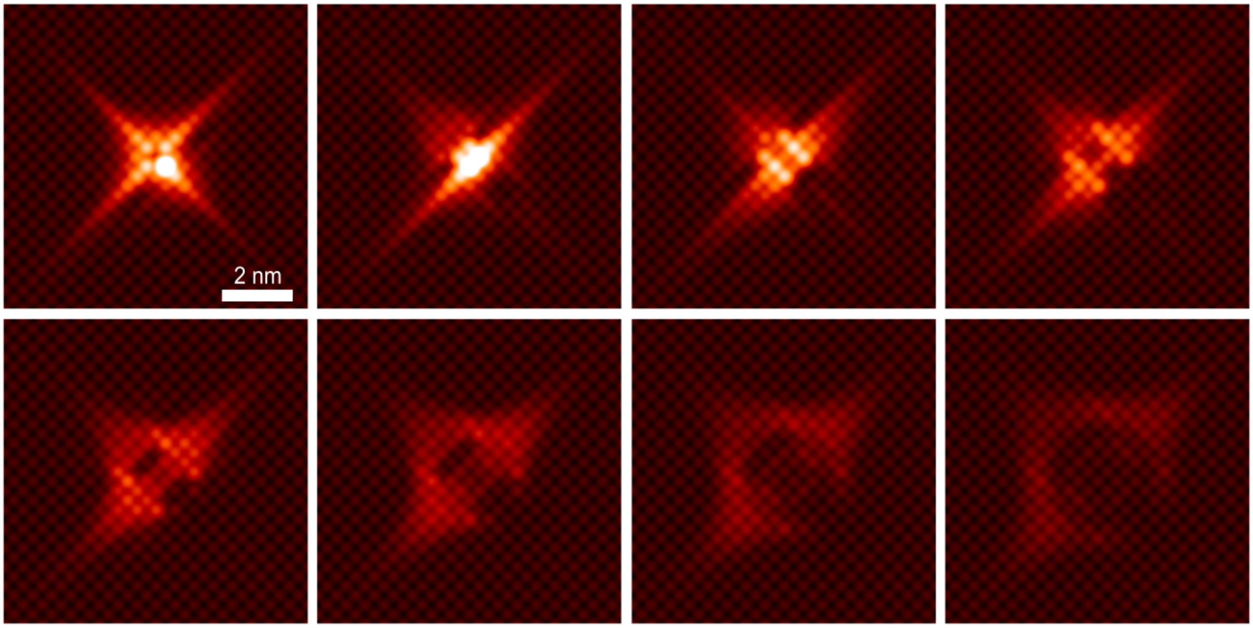

Figure S8: Series showing the acceptor spatial structure as a function of layer depth for a split-vacancy defect. The spatial structure is skewed towards the upper-left quadrant, reflecting the strong S_6 site symmetry. The layer spacing along (001) is 1.357Å.

References

[1] Averkiev, N., and Il'Inskii, S. Y. Spin ordering of carriers localized at two deep centers in cubic semiconductors. *Physics of the Solid State 36*, 2 (1994), 278–283.

[2] Baldereschi, A., and Lipari, N. O. Spherical model of shallow acceptor states in semiconductors. *Physical Review B 8*, 6 (1973), 2697.

[3] Chuang, S. L. Efficient band-structure calculations of strained quantum wells. *Phys. Rev. B 43* (Apr 1991), 9649–9661.

[4] Çelebi, C., Garleff, J. K., Silov, A. Y., Yakunin, A. M., Koenraad, P. M., Van Roy, W., Tang, J.-M., and Flatté, M. E. Surface induced asymmetry of acceptor wave functions. *Phys. Rev. Lett. 104* (Feb 2010), 086404.

[5] Siegl, M. *Atomic-scale investigation of point defect interactions in semiconductors*. PhD thesis, UCL (University College London), 2018.

[6] Yakunin, A., Silov, A. Y., Koenraad, P. M., Wolter, J., Van Roy, W., De Boeck, J., Tang, J.-M., and Flatté, M. E. Spatial Structure of an Individual Mn Acceptor in GaAs. *Phys. Rev. Lett. 92*, 21 (may 2004), 216806.

[7] Ziegler, J. F., Ziegler, M. D., and Biersack, J. P. SRIM - The stopping and range of ions in matter (2010). *Nuclear Instruments and Methods in Physics Research B 268* (June 2010), 1818–1823.
